# Landscape barriers to pollen and seed flow in the dioecious tropical tree *Astronium fraxinifolium* in Brazilian savannah

**DOI:** 10.1371/journal.pone.0255275

**Published:** 2021-08-02

**Authors:** Ricardo O. Manoel, Bruno C. Rossini, Maiara R. Cornacini, Mário L. T. Moraes, José Cambuim, Marcelo A. M. Alcântara, Alexandre M. Silva, Alexandre M. Sebbenn, Celso L. Marino

**Affiliations:** 1 Instituto de Biotecnologia/ UNESP, Botucatu, São Paulo, Brazil; 2 Instituto de Biociências/ UNESP, Botucatu, São Paulo, Brazil; 3 Faculdade de Engenharia de Ilha Solteira/UNESP, Ilha Solteira, São Paulo, Brazil; 4 Departamento de Melhoramento e Conservação Genética, Instituto Florestal de São Paulo, Piracicaba, São Paulo, Brazil; Austrian Federal Research Centre for Forests BFW, AUSTRIA

## Abstract

Gene flow studies provide information on gene exchange between populations, which is essential for developing genetic conservation strategies. Such analyses enable a better understanding of the life history and seed and pollen dispersal mechanisms of plant species. In this study, we investigate pollen and seed flow in a regenerant population of the pioneer species *Astronium fraxinifolium* in an area degraded during the construction of a hydroelectric dam. We mapped, sampled, sexed, and genotyped 386 individuals in the regenerant population (RP), as well as 128 adult trees located along two highways adjacent to the degraded area; one in Mato Grosso do Sul State (MS) and other in São Paulo State (SP). Parentage analyses was carried out for 370 individuals of the RP population, using as putative parents 348 individuals from RP and all 128 individuals sampled in MS and SP. Based on parentage analysis and eight microsatellite loci, our analyses revealed that for individuals of the RP with an identified father (pollen donor), 1.1% of the pollen was dispersed up to 532 m, while for those with an identified mother (seed donor), 0.5% of seeds were dispersed up to 4,782 m. However, a large proportion of pollen (76.5%) and seeds (57%) immigrated from trees outside the sampled populations. Pollen and seeds were dispersed through a pattern of isolation by distance. Genetic diversity was significantly similar between adults of both highway populations and individuals from RP, with significant levels of inbreeding detected only in RP. Our results demonstrate that the nearest trees contributed pollen and seeds for the recovery of the degraded area, indicating reproductive spatial isolation among the sampled populations due to the damming of the river. Such results help to understand the process of regeneration for *A*. *fraxinifolium* in regenerant populations to inform strategies for conservation and environmental recovery with this species.

## Introduction

Human interference in environments can trigger changes in biodiversity and disrupt ecosystem processes in tropical forests [1], especially in the savannah biome, one of the richest and possibly most threatened tropical savannas in the world [2]. For example, reductions in natural population size and tree density can lead to increases in levels of inbreeding and genetic differentiation among isolated forest remnants [3]. These factors can augment the risk of extinction for species and populations due to restrictions in gene flow dynamics and changes in mating patterns that can continue for several generations [3–7]. Thus, the outcome for a species that remains isolated will depend on its ability to persist, despite the size of the remaining reproductive population (bottleneck effect), restrictions in gene flow, genetic drift, and increases in self-fertilization and mating between relatives, which generally result in a decrease in genetic diversity [8–10] and accentuate inbreeding in descendant populations, where the inbreeding can result in inbreeding depression (decrease in survival, adaptation, and growth vigor) [11–16].

However, many tropical tree species are resilient to the effects of spatial population isolation and respond to genetic pressures through mechanisms of long-distance gene flow, long life span, and flexible mating systems [3]. Gene flow studies are a powerful tool that can inform the conservation and management of species and genetic resources. Some tropical tree species can be resilient to the effects of spatial isolation due to forest fragmentation if the distance between the remaining populations is within the range across which pollen and seed vectors can travel. Some studies have shown that extensive gene flow through pollen and/or seeds at distances greater than 10 km can maintain connectivity, but these events are rare [3, 17–21]. In general, gene flow occurs at distances of less than 1 to 5 km, with seed dispersal commonly occurring at shorter distances than pollen dispersal [17, 20–22]. Thus, the genetic resilience of fragmented populations depends on the degree of spatial isolation and the distance over which pollen and seeds are dispersed. Other factors influencing genetic resilience are the fact that many species present a long life span (some can live more than 100 year) and generations overlap, which can maintain the remaining genetic diversity within populations for many years after forest fragmentation [3]. Meanwhile a flexible mating system can circumvent issues related to reproduction for some self-incompatible species, allowing seed reproduction through self-fertilization [3]. In addition, gene flow if limited can lead to increase in genetic drift and inbreeding and reduced levels of genetic diversity, defining the patterns of spatial genetic structure (SGS) of the population. In this context, the gene flow also affects the effective population size, which is determined by the number and spatial distribution of the different pollen and seed donors (non-relatives and non-inbreeding) that contribute to the effective dispersion [20] and formation of the next generations.

Few have assessed the ecological genetic effects of Hydropower Plant (HPP) dam construction or rivers as barriers to pollen and seed dispersal among tree populations [23–25]. Such infrastructure projects are responsible for a significant amount of forest loss, creating forest fragments of different sizes and degrees of isolation due to the deforestation of areas for soil removal, transposition of the riverbed, and flooding of reservoirs. In addition, soil removal for the translocation process can include many soil layers, leading to the removal of nutrients and reducing the viability of plant maintenance in the degraded area. Quantifying the impact of habitat degradation on forest remnants not only helps to assess the consequences of these changes, but also contributes to the conservation of remnants and the development of effective and sustainable strategies for the management of genetic resources of species that inhabit degraded landscapes [21, 26–30].

In this context, this analysis focuses on an area used as a soil loan (considered herein as the regenerant population), where soil was removed to provide the foundation for the Ilha Solteira Hydroelectric Dam, in Selvíria, Mato Grosso do Sul, Brazil. As such, this landscape was transformed into a degraded habitat through the elimination of native vegetation of the Brazilian savannah, along with the means to support biotic regeneration, such as the seed bank, seedlings, regrowth, and associated microbiota and soil nutrients. However, some native savannah tree species, such as *Astronium fraxinifolium* Schott. (Anacardiaceae), emerged naturally in several of these inhospitable sites. This species has been previously reported as occurring naturally in totally degraded habitats, especially along road margins or in forest fragments [31]. This dioecious tree species, popularly known as “gonçalo alves”, is typical of the Brazilian savannah and is also found in the Amazon, Caatinga, Atlantic Forest, and Pantanal biomes. It is a pioneer tree that commonly occurs in anthropogenically degraded areas; as such, it is often used in environmental restoration. In deciduous forest remnants of the savannah, *A*. *fraxinifolium* occurs at a low density when compared to other species. It was listed as vulnerable to extinction in the past decades and more recently as data deficient [32, 33]. Reproduction has been observed at 18 years of age ande flowering phenology is annual and occurs between the months of July and October (16% of trees) [34]. Pollination is mainly mediated by bees; while seeds that do not separate from the drupe-shaped, light, small, and apiculate fruit, are wind dispersed, which is facilitated by their five-sepal star shape [35]. The seeds have antifungal, antitumor, antileishmanial and dermatological properties [36]. Trees can reach 80 cm in diameter at breast height (DBH) and 12 m in height. Its wood is heavy, with a density around 1.09g/cm³, highly durable, and used in naval and civil construction [37].

In this study, we used eight microsatellite loci and parentage analysis to analyze whether *A*. *fraxinifolium* individuals that emerged in the regenerant population (RP) are genetically related to distant individuals isolated throughout the landscape as a result of long-distance pollen and seed flow due construction of the dam, or if parentage is from trees located near to the soil loan area. It is worth noting that studies on pollen and seed dispersal patterns for *A*. *fraxinifolium* trees are absent from the literature. We specifically answered the following questions: *i)* Due to the spatial isolation caused by the widening of the river to form the HPP reservoir, do remnant populations equally contribute to the regenerant population? *ii)* What are the distance and dispersal patterns of pollen and seeds of trees established in the regenerant population; *iii)* Are there differences in the levels of genetic diversity and inbreeding between the remnant populations located along highways and the regenerant population? *iv)* What is the degree of relatedness (estimated by group coancestry coefficient) and the effective population size in the regenerant population?

## Materials and methods

### Study site and sampling

**Fig 1:**
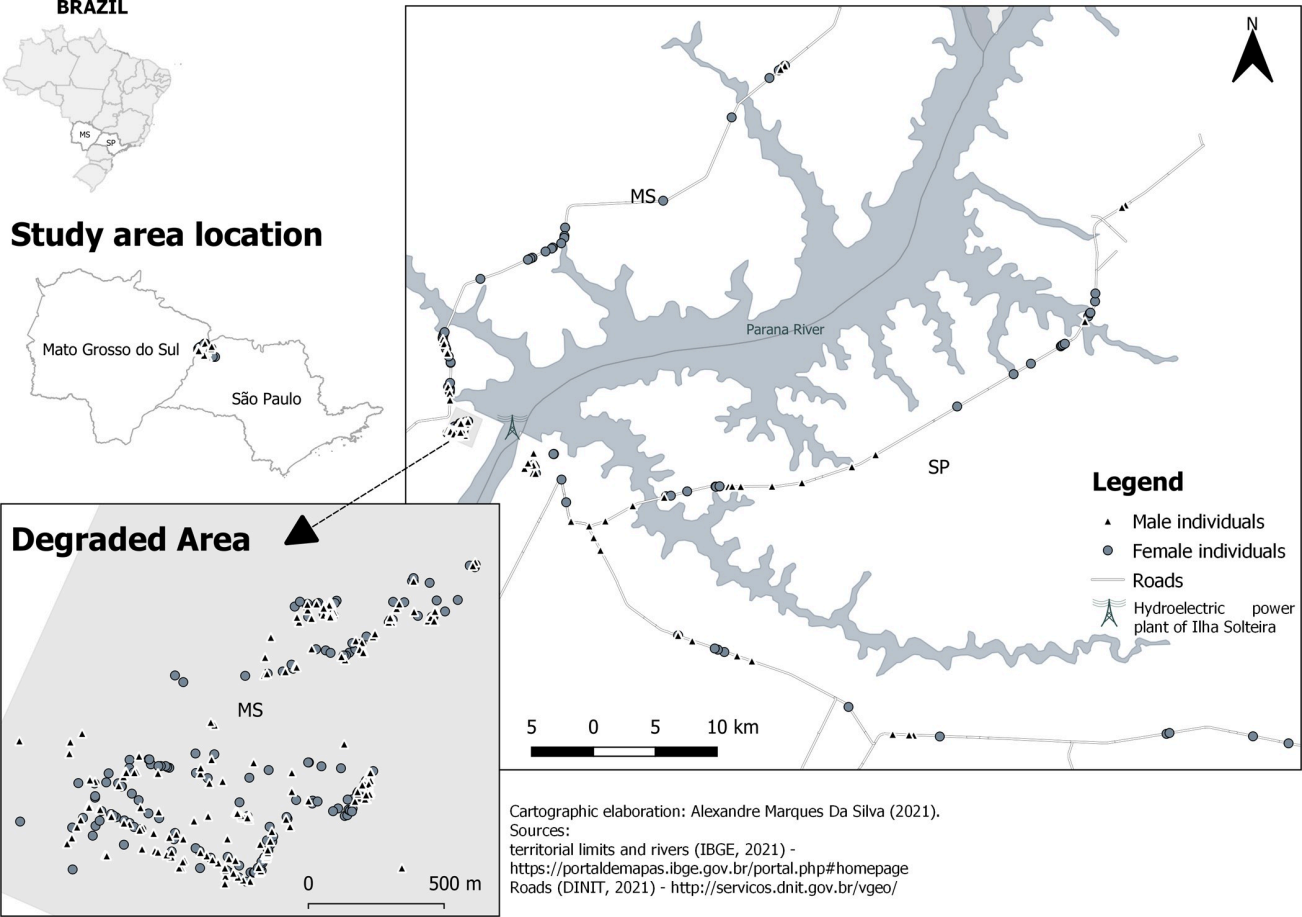
Spatial distribution of *Astronium fraxinifolium* individuals in the Regenerant Population (RP) and in the states of Mato Grosso do Sul (MS) and São Paulo (SP), Brazil.

Collection was conducted in a savannah area with significant soil and vegetation degradation in Selvíria, Mato Grosso do Sul, Brazil, located on the Paraná River (Fig 1; 20°22’26.4" S; 51°24’06.9" W). The climate of the region is tropical with dry winters, humid summers, average annual precipitation of 1,354 mm, and average temperature of 24.5° C. The site of the regenerant population (RP) was used as a soil loan for the construction of the Ilha Solteira Hydroelectric Dam built in 1968 and is located on the border between São Paulo (SP) and Mato Grosso do Sul (MS) States. An 8.6 m layer was removed from the original soil profile for the construction of the dam foundation. Therefore, returning the site to its original conditions is extremely long and difficult due to poor soil conditions. Collection of leaf tissues was authorized by the Institute for Biodiversity Conservation (ICMBio), linked to the Ministry of the Environment (MMA) under the number 73998–1.

To investigate the isolation effects of dam construction on pollen and seed flow in the RP population we also sampled two populations of isolated trees located along highways on both sides of the river, one in Mato Grosso, along highway BR158 between the municipalities of Selvíria and Aparecida do Taboado, and one in São Paulo, along highways SP310 and SP595, between the municipalities of Ilha Solteira, Sud Mennucci, and Santa Fé do Sul. After exhaustive sampling along the highways of the region, material collected from these isolated trees were used to install an experiment in 1998 at the experimental farm belonging to the Faculdade de Engenharia—UNESP—Ilha Solteira Campus, in Selvíria. The MS and SP populations are approximately 10 km apart, but occur in different biomes, one in the Semideciduous Latifoliate Forest (SP) and the other in the savannah (MS). Beyond the three areas sampled in this study, the species occurs commonly throughout the region. For DNA analysis, we collected leaves from individuals at the three sites that were georeferenced (using a GPS III-Garmin, USA), measured for diameter at breast height (DBH) and plant height (H, only in RP population), and sexed by visual inspection of flowers (S1 Table). In RP, all 386 identified individuals (2.27 trees/ha) within the plot were sampled (leaves collected with the help of a trimmer; DBH = 21.7 cm; H = 10.1 m; 180 females and 206 males; mean distance = 243 m, ranging from 1–928 m). In MS, 49 individuals were sampled (DBH = 31.33 cm; 37 females and 12 males; mean distance = 17,209 m; ranging from 4–58,777 m) and in SP 79 individuals were sampled (DBH = 30.28 cm; 39 females and 40 males; mean distance = 14,240 m; ranging from 2–57,287 m). All sample sites have undergone significant fragmentation that likely occurred at the same time. However, most trees in the RP population (75.2%) present DBH≤ 25 cm, whereas in MS and SP only 30.1 and 38% of trees, respectively, have a DBH≤ 25 cm, indicating that the population is composed predominantly of individuals that are younger than those in the MS and SP populations (S1 Fig), and were established after dam construction. Furthermore, based on the estimate of the mean annual increment (MAI) for DBH at 18 years of age for the *A*. *fraxinifolium* provenance and progeny test established in the same area as RP population (DBH/age = 11.45/18 = 0.636 cm/year [37]), we can speculate that 75% of trees in RP are younger than 40 years of age, and were established post-fragmentation, whereas in MS and SP more than 60% of individuals are older than 40 years. Considering that individuals in MS and SP are generally older than those in RP, the parentage analyses for RP included older trees from all three populations as putative pollen (father) and ovule (mother) parents.

### Microsatellite genotyping

Genomic DNA was isolated from fresh leaves using the protocol described in [38]. Eight dinucleotide microsatellite loci (Ga02, Ga03, Ga04, Ga05, Ga06, Ga07, Ga08, and Ga09) were amplified according to Cornacini et al. (2021) [39]. Some individuals used in Cornacini et al. were included in this analysis to complement the sampled populations (30 trees from MS and 30 from SP). Amplified PCR products were run on an ABI3130XL automatic DNA sequencer (Applied Biosystems) with the GeneScan 500 LIZ size standard and analyzed in the GeneMapper Software 5.0 (Applied Biosystems).

### Analysis of genetic diversity and population structure

The frequency of null alleles and corrected fixation inex for null alleles were estimated using the INEST 2.0 software [40]. Genotypic linkage disequilibrium between pairwise loci of MS, SP, and RP populations was estimated using the FSTAT software [41]. This software was also used to calculated the genetic diversity for each population for the indices: the total number of alleles across all loci (*K*), allelic richness (*R*), and observed (*H*_*o*_) and expected (*H*_*e*_) heterozygosity, as well as to estimated the fixation index (*F*) and its statistical significance was calculated by the permutation of alleles among individuals. The genetic differentiation $\left(G_{ST}^{'}\right)$ among all populations and between pairwise populations was estimated using the method proposed for microsatellite loci [42]. The 95% standard error (1.96SE) for $G_{ST}^{'}$ was estimated among loci. To verify if these indices are significantly different between the populations, we used a jackknife resampling test among loci.

Population structure analysis was inferred with Bayesian analysis using the STRUCTURE software [43, 44], assuming an admixture model and correlated allele frequencies. We tested each K value (ranging from 1–10) with 20 independent runs, a burn-in period of 100,000, and 1,000,000 generations. The identification of the optimal K was inferred using the method outlined in the Structure Harvester software [45], based on delta K [46]. Additionally, we conducted a Principal Components Analysis (PCA), another multivariate method used to infer genetic variation using ´gstudio´ package [47] in the R software environment.

### Analysis of spatial genetic structure and effective population size

We estimated the spatial genetic structure (SGS) and the effective population size (*N*_*e*_) only in the RP population, where all individuals from the site were sampled. Although we have exhaustively sampled individuals in the other two sample populations, this has only been done along the highways, and probably other individuals of the species may exist in these locations. Thus, to avoid speculation, these analyzes were not carried out. The analysis of SGS was carried out for the RP population based on estimates of the coancestry coefficient (*θ*_*xy*_) [48], using the SPAGEDI software [49]. To visualize SGS, seven distance classes (10–25, 25–50, 50–75, 75–100, 100–250, 250–400, and 400–928 m) were used. We obtained the statistical significance of *θ*_*xy*_ by comparing the confidence interval limits at a 95% probability for the average estimated *θ*_*xy*_ for each distance class, as calculated by the permutation of individuals among distance classes (1000 permutations). We also estimated the standard error of the mean *θ*_*xy*_ values using jackknife resampling between loci. In addition, the classes of maximum and average distances, number of pairs of individuals, percentage of participation of individuals, coefficient of variation of participation of individuals and mean and standard error of *θ*_*xy*_ values by distance classes, were presented in a table, similar to presented in the study by Browne and Karubian [50]. To compare the strength of SGS with other studies, the *Sp-*statistic [51] was calculated by, *Sp* = -*b*_*k*_/(1-*θ*_1_), where *b*_*k*_ is the slope of the regression of the *θ*_*xy*_ values on the natural logarithm of spatial distance and *θ*_1_ is the mean pairwise coancestry coefficient estimate in the first distance classe (10 m). The standard error of *b*_*k*_ was calculated using jackknife resampling between loci.

The group coancestry (Θ) for RP population was estimated by hand,

$$\Theta =\frac{\sum_{i=1}^{n_{m}}\sum_{j\neq i}^{n_{m}}\theta_{ij}}{4n_{m}^{2}}+\frac{\sum_{i=1}^{n_{f}}\sum_{j\neq 1}^{n_{f}}\theta_{ij}}{4n_{f}^{2}}+\frac{\sum_{i=1}^{n_{m}}\sum_{j=1}^{n_{f}}\theta_{ij}}{2n_{f}n_{m}},$$

where *n*_*m*_ and *n*_*f*_ are the number of males and females, respectively, as described for dioecious species [52] and the effective population size (*N*_*e*_) was estimated by,

$$N_{e}=\frac{0.5}{\Theta \left(\frac{n-1}{n}\right)+\frac{1+F}{2n}},$$

where *n* is the sample size (*n* = *n*_*m*_ + *n*_*f*_) [53].

### Parentage analysis

The analysis of realized pollen and seed dispersal for 370 RP individuals (DBH< 40 cm) was carried out using the CERVUS 3.0.7 software [54]. This software was also used to calculate the combined non-exclusion probability for the first parent (*P*_1_), combined non-exclusion probability for the parent pair (*P*_2_), and combined non-exclusion probability of identity for two unrelated individuals (*Q*_*i*_). The parentage analyses was carried out comparing seedlings genotypes with putative mother (females) and father (males) genotypes candidates of the, using CERVUS 3.0.7 software based on the Δ statistic, which is the difference between the LOD scores of the two most probable candidate pollen parents (54). To find the critical value of Δ for strict (95%) and relaxed (80%) confidence levels in the parentage analyses, simulation was carried out using 10.000 repetitions, 0.01 as the proportion of loci mistyped, 370 of candidate mothers and 370 of candidate fathers and 70% of proportion of sampled mother and father parents and the minimum number of loci required for parentage analysis was set to six. All females and males trees of the three populations (RP, MT, and SP) were used as putative mother and father candidate parents for each RP seedlings. If a candidate male had an Δ exceeding the critical value of Δ, determined from simulations, it was considered a true potential parent. If a seedling had no potential mother parent in the RP population, it was considered as seed immigrant; if a seedling had no father parent within RP population it was assumed that the mating event had the participation of pollen immigration from outside the site. The analysis was carried out as follows: 1) due to the missing genotypes in the samples of RP (18 female and 15 male individuals with one missing genotype and 4 female and 3 male individuals with two missing genotypes), MS (2 female and 2 male individuals with one missing genotype and 2 females with four missing genotypes), and in SP (1 male individual with two missing genotype), parentage was only accepted if a minimum of six pairs of loci were compared between assigned RP individual and putative sib-mother, sib-father, or for the sib-mother-father trio. The two females of the MS population with four missing genotypes were excluded as putative mothers; 2) to be conservative and due to the fact that 16% of trees were reported as flowering at 18 years of age [39], the assigned female and male parents to RP individuals were accepted if the difference between sib and parent age was at least 13 years. The age of all trees was estimated based on the mean annual increment for DBH (DBH/age = 11.45/18 = 0.636 cm/year), as noted above [39]. All females (164) and males (184) with DBH ≥ 7 cm (estimated age > 14 years) within RP, as well as those in the MS (females = 35; males = 12) and SP (females = 39; males = 40) populations, were used as putative ovule and pollen parents of individuals within RP. Individuals assigned neither female nor male trees within RP were designated as originating from seed immigration. Those not assigned to any female were determined as originating from realized seed immigration, and those not assigned to any male were determined as originating from realized pollen immigration. The spatial positions (x and y coordinates) of individuals within RP and the assigned ovule donors (mother) were used to estimate the mean, standard deviation (SD), median, minimum, and maximum realized seed dispersal distances. For individuals within RP that were assigned both female and male parents, the spatial positions (x and y coordinates) of the mother and father were used to estimate the mean, SD, median, minimum, and maximum realized pollen dispersal distances. To determine if the realized seed dispersal distance and frequency of assigned individuals to female trees, and if the pollen dispersal distance (distance between assigned female and male) and frequency of assigned individuals to both female and male trees were correlated, the Pearson coefficient of determination (*R*^2^, linear) was used. To determine if female and male fertility are associated with their respective DBH and H, we used the Spearman’s rank correlation coefficient (*ρ*).

## Results

### Genetic diversity and structure

Our results show that *A*. *fraxinifolium* can reach a height of 19.8 m (S1 Table). For the entire sample of trees from all populations (*n* = 514), a total of 122 alleles were found in the eight studied loci, ranging among populations from 86 to 101 alleles (Table 1). The RP population presented the greatest number of private alleles (*P*_*a*_ = 9), followed by SP (*P*_*a*_ = 8) and MS (*P*_*a*_ = 5). Significant genotypic linkage disequilibrium (LD) was found only in RP between pairwise loci Ga02xGa05, Ga03xGa05, Ga05xGa08, Ga06xGa07, Ga06xGa08, and Ga07xGa08. Given that LD may be the result of inbreeding, bottleneck or founder effects, and was only detected in one of our sample populations, we continued the analyses using all eight loci. Based on a jackknife test, the mean allelic richness (*R*), expected heterozygosity (*H*_*e*_), and fixation index (*F*) were significantly higher in MS than the other populations (Table 1). The indices *R*, *H*_*e*_, and *F* were also significantly higher in SP than RP. The fixation index (*F*) was positive and significantly (P< 0.05) higher than zero in RP (0.079), suggesting biparental inbreeding. The frequency of null alleles was low (< 0.1) in all loci of the three populations (Tables 1 and S2). The genetic differentiation $\left(G_{ST}^{'}\right)$ was higher among all populations (0.580 ± 0.176, mean ± 1.96xstandard error), between (RP+MS) x SP (0.566 ± 0.176, mean ± 1.96SE), and between RP x SP (0.566 ± 0.192, mean ± 1.96SE), indicating that a large proportion of the genetic diversity is distributed among populations. Meanwhile, the genetic differentiation between RP x MS (0.348 ± 0.162, mean ± 1.96SE) and MS x SP (0.368 ± 0.161, mean ± 1.96SE) indicate that a large part of the genetic diversity is distributed within populations.

**Table 1:** Results of mean genetic diversity for populations of *Astronium fraxinifolium* in the Regenerant Population (RP), and along highways of MS and SP.

	MS (1.96SE)	SP (1.96SE)	RP (1.96SE)
Sample size: *n*	49	79	386
Total number of alleles: *K*	101	86	91
Private alleles: *P*_*a*_	5	8	9
Allelic richness: *R*	12.5 (0.2)^a^	10.0 (0.2)^b^	9.1 (0.2)^c^
Observed heterozygosity: *H*_*o*_	0.843 (0.006)^a^	0.825 (0.005)^b^	0.731 (0.005)^c^
Expected heterozygosity: *H*_*e*_	0.851 (0.005)^a^	0.829 (0.004)^b^	0.793 (0.005)^c^
Fixation index: *F*	0.009 (0.003)^a^	0.005 (0.004)^a^	0.079* (0.004)^b^
Frequency of null alleles: *Null*	0.008	0.008	0.004
Fixation index corrected: *F*_*null*_	0.002 (0.018)^a^	-0.015 (0.022)^a^	0.073* (0.024)^b^

*R* is the allelic richness for 46 individuals genotyped for eight loci; 1.96SE is the standard error.

*P< 0.05; Different letters mean significant differences at 5% probability based on a jackknife test (among loci).

**Fig 2:**
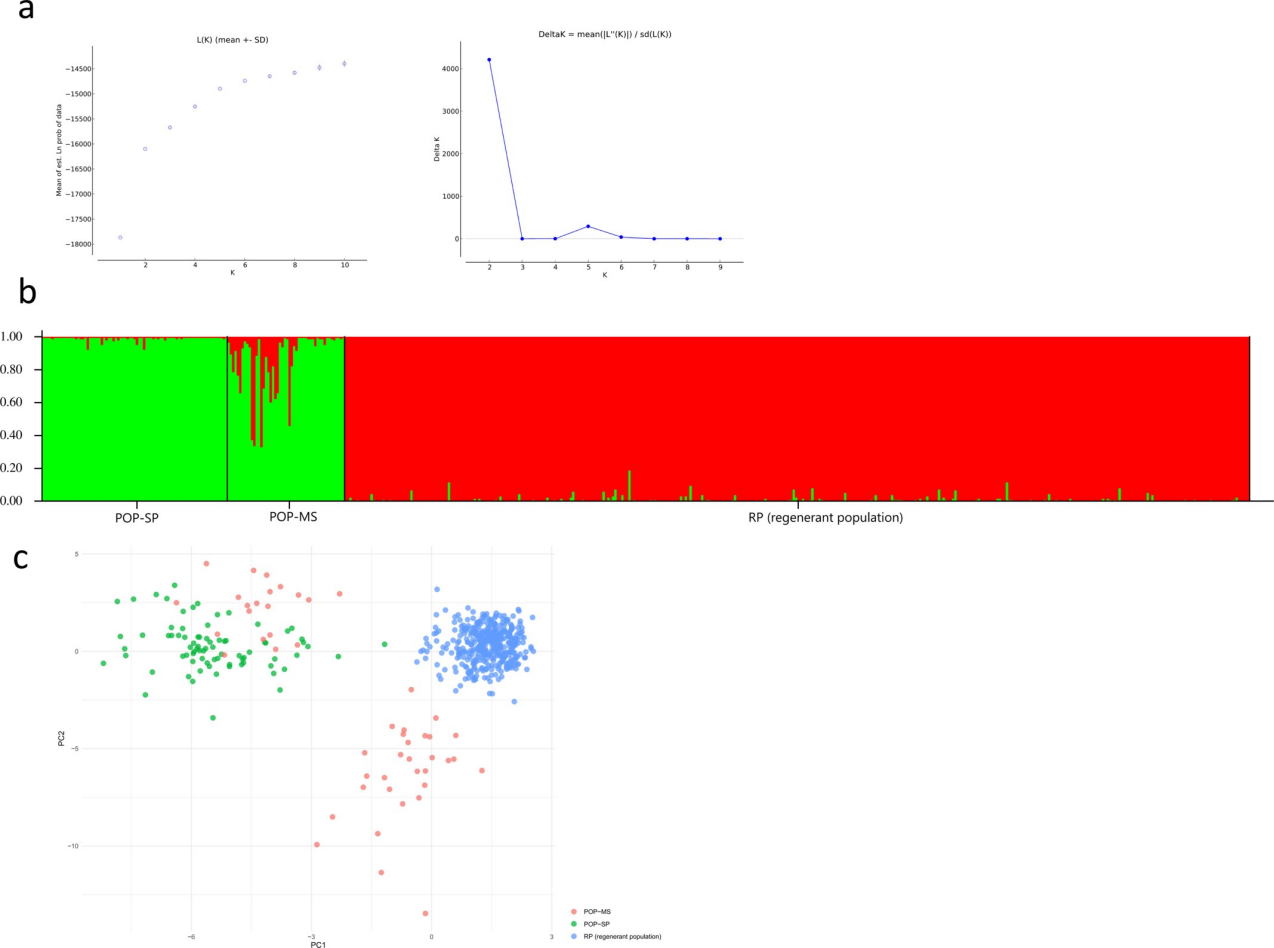
Population structure analysis of *Astronium fraxinifolium*. (a) Values of Ln P(k) and Delta (K) [48]. (b) STRUCTURE analysis showing two genetic clusters. (c) Principal component analysis.

The Bayesian analysis suggest that are two genetic clusters (K = 2) based on delta K analysis. However, the Ln P (k) plateau indicates the existence of more clusters, with some individuals from MS closely related to those in RP (Fig 2A and 2B). Principal component analysis suggest the individuals from SP are more related to MS (Fig 2C).

### Spatial genetic structure and effective population size in the RP population

**Fig 3:**
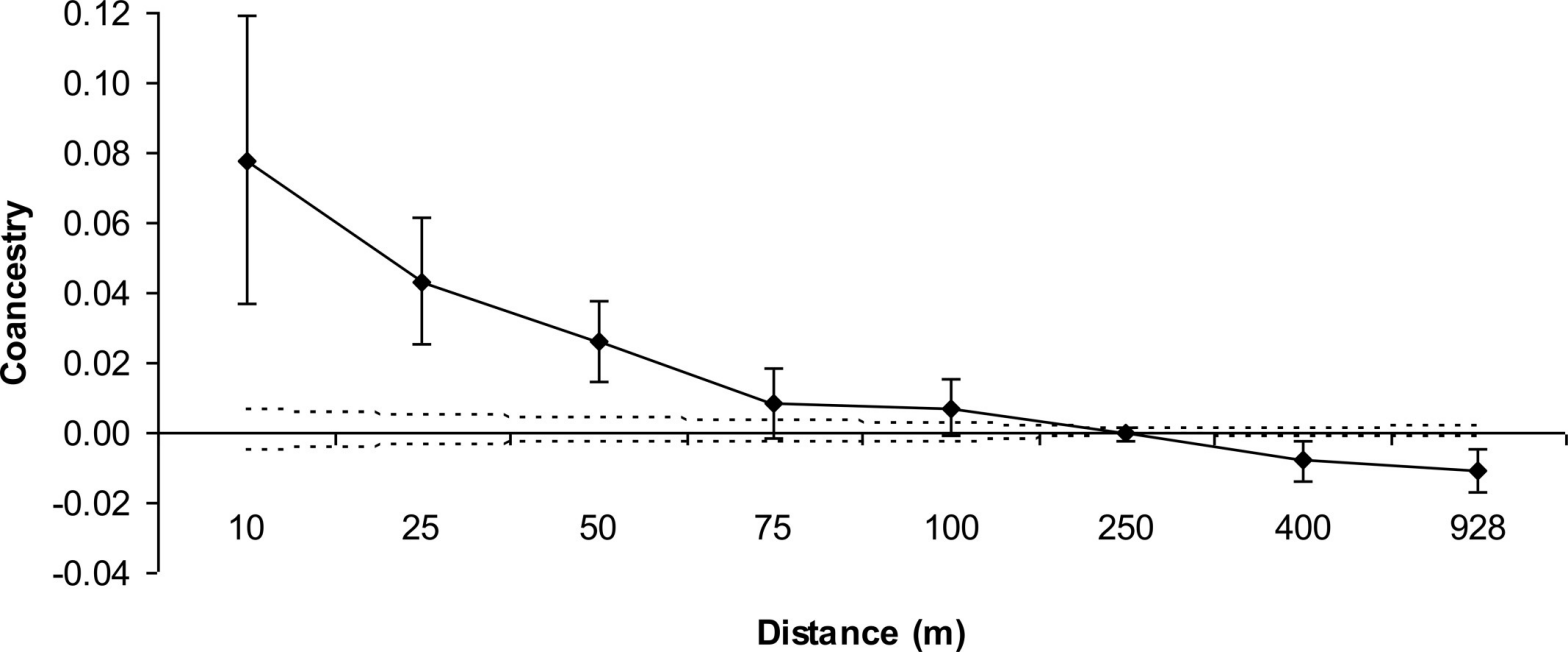
Spatial genetic structure of *Astronium fraxinifolium* in the Regenerant Population (RP). The continuous line represents the average estimated coancestry coefficient, and the dashed lines represent the confidence interval at 95% probability of the hypothesis of no SGS (*H*_*0*_: *θ*_*xy*_ = 0). The horizontal lines correspond to the standard error at 95% probability of mean *θ*_*xy*_ values.

Based on the confidence interval limits at a 95% probability for the average estimated *θ*_*xy*_ for each distance class, as calculated by the permutation of individuals among distance classes indicates higher SGS (175 m) than the results based on the confidence interval limits at a 95% probability, calculated by the standard error of the mean *θ*_*xy*_ values, using jackknife resampling between loci (65.2 m), suggesting relatedness between trees within this distance (Fig 3 and S3 Table). Since the results of the permutations were based on a larger number of resampling units (1000) than the results based on the jackknife resampling (8 loci), we were decided to use the results of the permutation for further discussion and recommendations. The intensity of SGS measured by the *Sp*-statistic was low 0.017 ± 0.007 (mean ± 1.96SE). The mean pairwise coancestry was low between females (*θ*_*f*_ = 0.000033), males (*θ*_*m*_ = 0.000365), and females and males (*θ*_*fm*_ = 0.001776), resulting in a low group coancestry (Θ = 0.002174) and suggesting that low levels of inbreeding would be expected in random mating (< 0.1%). The effective population size (*N*_*e*_) indicates that the 386 individuals of RP correspond to 144 non-inbred and unrelated trees in a population with random mating (*N*_*e*_/*n* = 0.38).

### Pollen and seed flow

**Fig 4:**
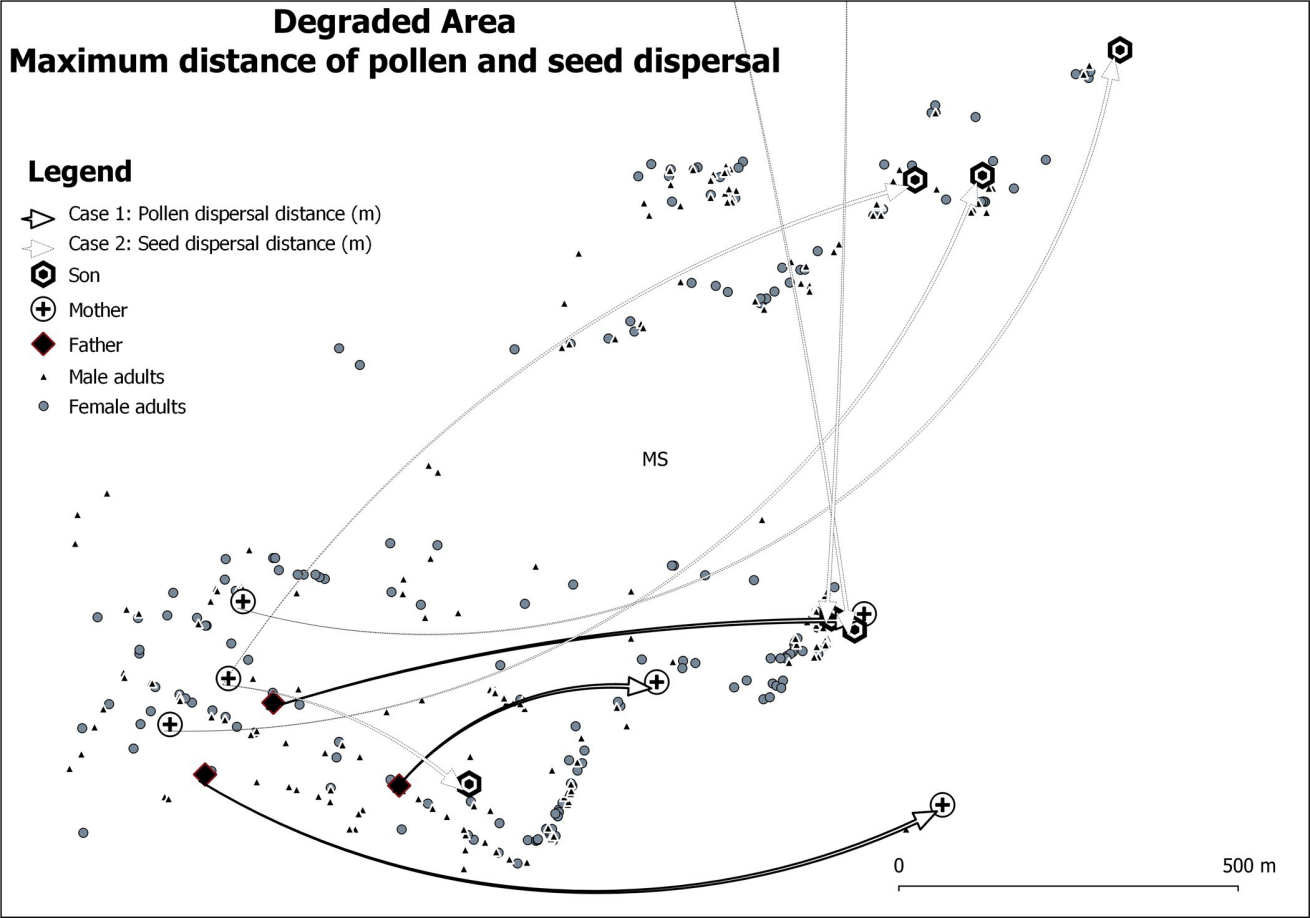
Spatial patterns of parentage identified in the degraded area. Arrows indicate the direction of the most distant identified pollen and/or seed donors.

The combined non-exclusion probability for the first parent (*P*_1_), combined non-exclusion probability for the parent pair (*P*_2_), and combined non-exclusion probability of identity for two unrelated individuals (*Q*_*i*_) were low (0.0022406, 0.0000001, and 0.00000000001, respectively), indicating that the eight loci present levels of polymorphism with sufficient resolution for parentage analysis. A putative mother (ovule donor) was found for 43% of the individuals in RP, with 42.5% located within RP and 0.5% originating from females in MS. However, 211 seedlings were not assigned to any female in our sample, indicating a total of 57% of seeds originating from females possibly located outside the RP population (Table 2 and Fig 4 and S4 Table). A putative father (pollen donor) was found for 23.5% of the individuals in RP, all located within RP. No assigned pollen donor from within our sample was identified for 283 of seedlings in RP, indicating a total realized pollen immigration rate of 76.5% from outside the RP population. Both ovule and pollen donors were assigned for 17.3% of individuals in RP, with all located within RP. No assigned parent pair within our sample was identified for 306 seedlings, indicating a total of 82.7% of both ovule and pollen immigration from outside the RP population.

**Table 2:** Results of parentage analysis for individuals of the regenerant population (father or pollen parent; mother or seed parent) for *Astronium fraxinifolium*.

	Seeds (Mother)	Pollen (Father)	Pollen + Seeds
Sample size: *n*	370	370	370
Total assigned (%)	159 (43)	87 (23.5)	64 (17.3)
Assigned within RP (%)	157 (42.5)	87 (23.5)	64 (17.3)
Assigned from MS (%)	2 (0.5)	0	0
Assigned from SP (%)	0	0	0
Total immigrant (%)	211 (57)	283 (76.5)	306 (82.7)
Mean dispersal distance (m)	183 ± 67	185 ± 28	205 ± 35
Median dispersal distance (m)	77	163	165
Min/max dispersal distance (m)	1/4782	1/532	5/532

± is the 95% standard error (1.96SE)

**Fig 5:**
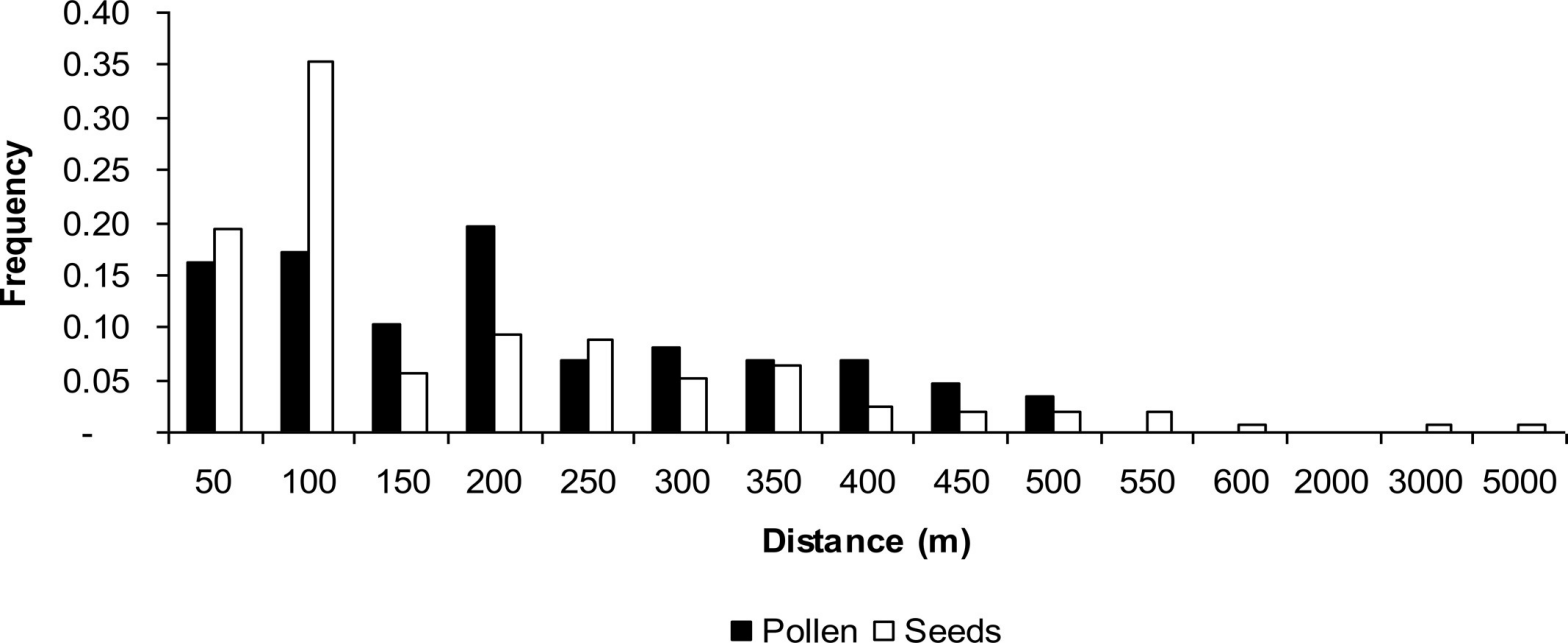
Pollen and seed dispersal distance for *Astronium fraxinifolium* individuals in the Regenerant Population (RP).

The realized pollen dispersal distance ranged from 1 to 532 m, with a mean of 185 m and median of 163 m. Realized seed dispersal distance ranged from 1 to 4,782 m, with a mean of 183 m and median of 77 m (Table 2 and Fig 5). The distance between assigned mother and father parents explains 45.3% (*R*^2^ = 0.453, *P*< 0.01) of pollen dispersal distance, while 40.6% (*R*^2^ = 0.406, *P*< 0.01) of seed dispersal distance is explained by the distance between mothers and their offspring. Furthermore, the Spearman’s rank correlation coefficient (*ρ*) was significantly positive between the number of assigned individuals per female tree and female DBH (*ρ* = 0.384, *P*< 0.001), and for female height (*ρ* = 0.306, *P* = 0.023). Significantly positive correlation was detected between the number of assigned individuals per male tree and male DBH (*ρ* = 0.431, *P*< 0.002) and height (*ρ* = 0.415, *P* = 0.007).

## Discussion

Herein, we analyze the relationship between individuals from the regenerate and two remnant populations located along highways in São Paulo and Mato Grosso do Sul and trees sampled from RP, respectively. The aim was to investigate the genetic composition of individuals that emerged in an area degraded due to the construction of a hydroelectric dam and assess the effects of spatial isolation on reproduction. Our goal was to understand the gene flow of this species across adjacent areas and determine the distance over which existing populations can contribute to the composition of new populations. In addition, we investigated pollen and seed dispersal distance and patterns in this totally altered environment. The two populations made up of spatially isolated trees occurring in the margins along highways in the states of SP and MS emerged in the period before the construction of the dam, given the need to construct the highways to reach the hydroelectric facility. Our results reveal important information about evolutionary processes of the species that only emerge after the degradation of the landscape, including long-range pollen and seed dispersal in a pattern of isolation by distance within RP and due to the barrier (river) between RP and SP populations. We found that the dam’s reservoir can act as a possible barrier for pollen and seed dispersal, since our data shows little interaction between SP and especially RP populations. Lastly, these results have important practical applications for environmental reforestation plans and *in situ* and *ex situ* genetic conservation programs.

### Genetic diversity and structure

The three studied populations present high levels of genetic differentiation ($G_{ST}^{'}$ ranging from 0.566–0.580), indicating that most of the genetic diversity is distributed among populations. However, $G_{ST}^{'}$ between RP x MS (0.348) and MS x SP (0.368) indicate that a large amount of the genetic diversity is distributed within populations. The long distances between the populations, along with the history of fragmentation, can explain these differences. The isolated trees along the highways in MS and SP present high levels of genetic diversity and not significant inbreeding, whereas the trees in RP presented lower levels of genetic diversity and moderate levels of inbreeding (*F* = 0.07). The SP population is spatially isolated from MS and RP by the Paraná River. As such, pollinators are less likely to transport pollen between the two populations. In terms of seed dispersal, although the river has been widened and other anthropogenic disturbances have occurred in the region, it was expected that trees in SP would have promoted genetic connectivity across the landscape, with the possibility of pollen and seed dispersed to the RP. However, connectivity between SP and RP was not detected. Considering that MS and RP populations are in the same biome (Brazilian savannah), these similarities could be related to adaptation to similar conditions, in contrast to the vegetation, soil, and precipitation on the other side of river in the SP population. In a study of tree flora over time after habitat loss and fragmentation in the Atlantic Forest, the results indicated that the tropical flora is susceptible to taxonomic homogenization after severe degradation [55]. Thus, at the genetic level, this homogenization may be occurring for the species tested herein, since the genetic differences can be reduced within each biome as a result of the evolutionary process of adaptation to environment.

Another aspect to consider is the survival, maintenance, and predominance of *A*. *fraxinifolium* trees in the RP, with possible occurrence of genetic drift leading to the fixation of some favorable alleles in the regenerating population that resulted in the detected inbreeding depression. This can lead to adaptation to significant anthropogenic disturbances. Thus, despite finding an unfavorable habitat, genetic adaptation mechanisms may be taking place that enable the establishment and maintenance of populations as an alternate way for the species to survive the environmental consequences of human activities.

### Causes of spatial genetic structure

Spatial genetic structure (SGS) of genomic variation between populations in degraded landscapes is considered a key factor in reproductive biology. The ability of plants to expand their geographical distribution and maintain genetic diversity depends on pollen and seed dispersal vectors over short and long distances [56–58]. Seed dispersal by wind tends to produce more regular seed distribution [23] and this is an important factor affecting the occurrence of spatial genetic structure. For *A*. *fraxinifolium*, SGS is likely due to the combination of the distances of pollen and seed dispersal. Seed dispersal reached long distances (up to 4,781 m) and are dispersed by wind, as discussed above. Bees are the main vector of pollen dispersal [59], which have been shown to frequently travel long distances between trees, for example for *Tabebuia aurea* dispersing pollen up to 2.6 km [60] and up to 16 km for *Sorbus domestica* [61].

Spatial genetic structure of plant species has been compared with gene dispersal distances and climatic factors [17], suggesting that the mean values of the *Sp-*statistic do not differ between temperate and tropical regions. However, the authors did observe differences between wind- and animal-pollinated species in temperate regions and among seed dispersal vectors for species from tropical regions. Plant species pollinated by animals show higher *Sp*-statistic values than those pollinated by wind. In this study, the strength of SGS for individuals in RP, as shown through the mean *Sp* value (0.017), was similar to that found for species with pollen dispersed by animals (*SP* = 0.0171), but lower than that reported for species with insect pollination and seeds dispersed mainly by animals, including *Carapa procera* (0.028), *Virola michelii* (0.015), and *Vouacapoua americana* (0.032) [23]. The results obtained in this study suggest that species with seeds dispersed by wind have lower-magnitude SGS than those with seeds dispersed by animals. The dispersion of seeds over short distances resulted in the strong SGS for *A*. *fraxinifolium* in RP due to the increase in average coancestry between pairs of proximal individuals, as also observed by De Oliveira et al. [62]. SGS is mainly supported by the occurrence of genetic drift [63], caused by the low density of reproductive adults near to RP, which could have resulted in a barrier for the dispersion of pollen and seeds [23] and high levels of inbreeding due to elevated levels of mating between relatives.

In addition, the results obtained herein suggest that seed collection aiming at conservation and reforestation programs of *A*. *fraxinifolium* should consider a minimum distance of 175 m between seed-trees in the RP, the distance at which the SGS was significantly higher than zero, to avoid collecting seeds from related mother trees. The collection of seeds from related trees decreases the *N*_*e*_ of progeny array samples as seeds originating from genetic related mothers will also be genetic related. Therefore, the more disturbed the environment, the greater the distance necessary for sampling. However, these values are higher than those observed for *Acacia aroma* which has pollen and seed dispersal by bees and mammals, respectively, in which minimum and maximum distances of 50 to 100 m were suggested [64].

### Effective population size in RP

For regenerant individuals in RP, the fixation index (*F*) indicate a deficiency of heterozygous individuals and, therefore, a reduction in genetic diversity. Biparental inbreeding has been reported in studies of tree species pollinated by animals and insects [10, 16, 62, 65–67]. Here, the observed biparental inbreeding can be due to founder effect [68] occurring during the colonization process after dam construction, associated to the low density of reproductive individuals along with SGS and the limited pollen dispersal distance in the study. However, if the *N*_*e*_ is similar to or higher than 70, there is a high evolutionary potential and the possibility that these individuals can maintain genetic diversity over at least ten generations [69]. The effective population size (*N*_*e*_) was smaller than the sample size (*n*) for individuals of the RP, mainly due to the occurrence of SGS; the occurrence of related individuals within populations increases the group coancestry and, consequently, decreases the *N*_*e*_. Caballero et al. [69] suggested a minimum *N*_*e*_ of 70 for *in situ* genetic conservation of populations with random mating. Individuals in RP presented a higher *N*_*e*_ (144), indicating that the effective population size of these individuals is sufficient for *in situ* conservation. But, the creation of ecological corridors linking the fragments can promote increases in gene flow and the *N*_*e*_ in degraded landscapes [70].

Finally, in terms of sustainable management of the area in question, it is important to highlight the possible risk of even greater isolation from individuals located along the highways in MS or with other individuals not included in this study. If gene flow through pollen is interrupted, the survival of individuals in the landscape may be affected.

### Contribution to restoration of the RP by pollen and seed flow

In general, we found that of the studied populations only MS contributed pollen and seeds to RP. This confirms the isolation of the sampled RP population due to spatial distance among populations, barriers (the river), or landscape resistance resulting from dam construction for both pollen and seeds. Consequently, there is an absence of gene flow from SP among the emergent *A*. *fraxinifolium* individuals in the RP. Furthermore, we can see distinct effects on the rate of gene flow through pollen and seed dispersal, corroborating the observations made by [71].

Herein, there is a possible contribution of both pollen and seed flow from trees located in MS, particularly in terms of seed flow, which helped establish the population in RP. This finding is significant because it demonstrates that seeds from trees along roads are able to establish new populations, unlike pollen which only carries half the number of alleles. Over the years of colonization, there is an accumulation of migrant seeds, thus forming a new population before the regeneration begins to reproduce, which promotes high levels of genetic diversity [72]. However, long-range pollen flow in degraded landscapes is conditional on the successful establishment of short-distance seed migration [59, 71]. In this sense, we observed *A*. *fraxinifolium* seeds dispersed up to 4.7 km (mean 183 m), which is greater than that observed for other trees in natural populations with seed dispersal by wind (0.71–3.8 km) including *Cariniana estrellensis* [31], *Fraxinifolium excelsior* [73], *Jacaranda copaia* [74], and *Myracrodruon urundeuva* [75]. Additionally, pollen dispersal reached up to 532 m (mean 185 m), a value greater than that observed for other bee-pollinated trees occurring in fragmented landscapes, such as *Foetidia mauritiana* (15–296 m) [29] Instances of long-distance seed dispersal for some individuals can also be attributed to human activities, such as dispersal by vehicles or movement between construction sites [76].

The population structure analysis based on STRUCTURE and PCA analysis showed that the main genetically related clusters are MS and RP, suggesting the existence of at least two genetic clusters. The STRUCTURE results suggest that MS and RP are more genetic similar than SP and RP populations. However, our results also demonstrate that there are different genetic clusters for which it is impossible to determine the genetic origin of the regenerant individuals in the RP. Furthermore, a large proportion of the pollen and seed donors to the RP were not identified. This suggests that there are considerable numbers of individuals of the species in the landscape that were not sampled due to restricted access (i.e., private properties around the sampling sites) or that the populations of *A*. *fraxinifolium* have different population dynamics coupled with the dispersion method, particularly for seeds. Therefore, there is a clear need for further in-depth studies in the region to verify the existence of nearby genetically structured populations. These results can also be attributed to the small number of markers analyzed herein when compared to high-throughput methods such as single nucleotide polymorphisms (SNP) that can increase the resolution of such analyses.

Finally, we suggest that management of areas affected by dam construction must be done with caution, particularly in terms of understanding the genetic mechanisms of species. For species with a dioecious sexual system, for example, seed dispersal is an important factor in the genetic dynamics of populations. Therefore, priority must be given to the conservation of all forest fragments and remnants that can contribute significantly to genetic variability and ensure the maintenance of the species into the future.

## Supporting information

S1 Fig Distribution of frequency for diameter at breast height (DBH) of *Astronium fraxinifolium* in the Regenerant Population (RP), Mato Grosso do Sul (MS), and São Paulo (SP) populations. (DOCX)

S1 Table Sample size, mean, Standard Deviation (SD), minimum, maximum (min/max), and median for distance, diameter at breast height (DBH), tree Height (H), and estimated age for sampled individuals of RP, MS, and SP populations. (DOCX)

S2 Table Results for genetic diversity, null allele frequency (*Null*), uncorrected fixation index (*F*), fixation index corrected for null alleles (*F*_*null*_), per locus and as a mean for all loci for the Regenerant Population (RP) and populations along highways of MS and SP. (DOCX)

S3 Table Results intrapopulacional genetic structure (SGS) of *Astronium fraxinifolium* in the Regenerant Population (RP). (DOCX)

S4 Table Results of parentage analysis for simulations and observed at strict (95%) and relaxed (80%) critical delta (Δ) for individuals of the regenerant population (father or pollen parent; mother and seed parent) for *Astronium fraxinifolium*. (DOCX)
