# Supplementary material for: Landscape barriers to pollen and seed flow in the dioecious tropical tree Astronium fraxinifolium in Brazilian savannah
Source: PLoS One. 2021 Aug 2;16(8):e0255275. doi: 10.1371/journal.pone.0255275 (PMC8336915; doi:10.1371/journal.pone.0255275)
Supplement: S1 Fig — (DOCX) [file pone.0255275.s001.docx]

Landscape barriers to pollen and seed flow in the dioecious tropical tree *Astronium fraxinifolium* in Brazilian savannah

Ricardo O. Manoel^1^, Bruno C. Rossini^1*^, Maiara R. Cornacini^2^, Mário L. T. Moraes^3^, José Cambuim^3^, Marcelo A. M. Alcântara^2^, Alexandre M. Silva^3^, Alexandre M. Sebbenn^4^, Celso L. Marino^1,2^

^1^Instituto de Biotecnologia/ UNESP, Botucatu, São Paulo, Brazil

^2^Instituto de Biociências/ UNESP, Botucatu, São Paulo, Brazil

^3^Faculdade de Engenharia de Ilha Solteira/ UNESP, Ilha Solteira, São Paulo, Brazil

^4^**Departamento de Melhoramento e Conservação Genética,** Instituto Florestal de São Paulo, Piracicaba, São Paulo, Brazil

*** Correspondence:**Corresponding Author
[bruno.rossini@unesp.br](about:blank)

**Journal: PLOSONE**

**S1 Fig. Distribution of frequency for diameter at breast height (DBH) of** *Astronium fraxinifolium* **in the regenerant population (RP), Mato Grosso do Sul (MS), and São Paulo (SP) populations.**
