# Supplementary material for: Landscape barriers to pollen and seed flow in the dioecious tropical tree Astronium fraxinifolium in Brazilian savannah
Source: PLoS One. 2021 Aug 2;16(8):e0255275. doi: 10.1371/journal.pone.0255275 (PMC8336915; doi:10.1371/journal.pone.0255275)
Supplement: S1 Table — (DOCX) [file pone.0255275.s002.docx]

Landscape barriers to pollen and seed flow in the dioecious tropical tree *Astronium fraxinifolium* in Brazilian savannah

Ricardo O. Manoel^1^, Bruno C. Rossini^1*^, Maiara R. Cornacini^2^, Mário L. T. Moraes^3^, José Cambuim^3^, Marcelo A. M. Alcântara^2^, Alexandre M. Silva^3^, Alexandre M. Sebbenn^4^, Celso L. Marino^1,2^

^1^Instituto de Biotecnologia/ UNESP, Botucatu, São Paulo, Brazil

^2^Instituto de Biociências/ UNESP, Botucatu, São Paulo, Brazil

^3^Faculdade de Engenharia de Ilha Solteira/ UNESP, Ilha Solteira, São Paulo, Brazil

^4^ **Departamento de Melhoramento e Conservação Genética,** Instituto Florestal de São Paulo, Piracicaba, São Paulo, Brazil

*** Correspondence:**Corresponding Author
[bruno.rossini@unesp.br](about:blank)

**Journal: PLOSONE**

**S1 Table. Sample size, mean, standard deviation (SD), minimum, maximum (Min/max), and median for distance, diameter at breast height (DBH), tree height (H), and estimated age for sampled individuals of RP, MS, and SP populations.**

|  |  | RP (SD) | MS (SD) | SP (SD) |
| --- | --- | --- | --- | --- |
| Sample size: $n$ | All | 386 | 49 | 79 |
|  | Female | 180 | 37 | 39 |
|  | Male | 206 | 12 | 40 |
| Mean distance (m) | All | 243 (138) | 14240 (17172) | 17209 (11828) |
|  | Female | 250 (155) | 12979 (16520) | 17298 (10693) |
|  | Male | 240 (137) | 16915 (18790) | 14516 (11261) |
| Min/max [median] distance (m) | All | 1/928 [233] | 1/57287 [6947] | 1/58777 [29798] |
|  | Female | 1/927 [235] | 13/57287 [6899] | 1/47240 [19965] |
|  | Male | 1/928 [234] | 42/41500 [2308] | 4/58777 [11791] |
| DBH (cm) | All | 21.7 (9.4) | 31.33 (8.65) | 30.28 ± 10.73) |
|  | Female | 22.75 (9.96) | 31.3 (8.67) | 30.23 (10.48) |
|  | Male | 20.73 (8.9) | 31.46 (8.99) | 30.34 (11.11) |
| Min/max [median] DBH (cm) | All | 7.3/63.7 [18.6] | 16.8/49.9 [29.4] | 8.6/55.7 [28.8] |
|  | Female | 8.2/59.7 [19.9] | 16.8/49.9 [29.4] | 11.6/55.7 [26.7] |
|  | Male | 7.3/63.7 [17.8] | 17.7/45.6 [29.4] | 11.1/54.9 [29] |
| Height: H (m) | All | 10.1 (4.1) | - | - |
|  | Female | 11.14 (3.1) | - | - |
|  | Male | 10.79 (3) | - | - |
| Min/max [median] H (m) | All | 2.9/19.8 [18.6] | - | - |
|  | Female | 4.1/19.8 [11.9] | - | - |
|  | Male | 3.9/19.6 [8] | - | - |
| Mean age (years) | All | 34 (15) | 50 (14) | 48 (17) |
|  | Female | 36 (16) | 50 (14) | 48 (17) |
|  | Male | 33 (14) | 50 (15) | 48 (18) |
| Min/max age [median] (years) | All | 12/100 [29] | 27/79 [47] | 14/88 [46] |
|  | Female | 13/94 [31] | 27/79 [47] | 19/88 [43] |
|  | Male | 12/100 [28] | 28/72 [47] | 14/87 [46] |

The age of all trees was estimated based on the mean annual increment for DBH (DBH/age= 11.45/18= 0.636 cm/year) reported at 18 years of age for an *A. fraxinifolium* provenance and progeny test established at the same site of RP population [45].
