# Supplementary material for: Landscape barriers to pollen and seed flow in the dioecious tropical tree Astronium fraxinifolium in Brazilian savannah
Source: PLoS One. 2021 Aug 2;16(8):e0255275. doi: 10.1371/journal.pone.0255275 (PMC8336915; doi:10.1371/journal.pone.0255275)
Supplement: S2 Table — (DOCX) [file pone.0255275.s003.docx]

Landscape barriers to pollen and seed flow in the dioecious tropical tree *Astronium fraxinifolium* in Brazilian savannah

Ricardo O. Manoel^1^, Bruno C. Rossini^2*^, Maiara R. Cornacini^2^, Mário L. T. Moraes^3^, José Cambuim^3^, Marcelo A. M. Alcântara^2^, Alexandre M. Silva^3^, Alexandre M. Sebbenn^4^, Celso L. Marino^1,2^

^1^Instituto de Biotecnologia/ UNESP, Botucatu, São Paulo, Brazil

^2^Instituto de Biociências/ UNESP, Botucatu, São Paulo, Brazil

^3^Faculdade de Engenharia de Ilha Solteira/ UNESP, Ilha Solteira, São Paulo, Brazil

^4^ **Departamento de Melhoramento e Conservação Genética,** Instituto Florestal de São Paulo, Piracicaba, São Paulo, Brazil

*** Correspondence:**Corresponding Author
[bruno.rossini@unesp.br](about:blank)

**Journal: PLOSONE**

**S2 Table. Results for genetic diversity, null allele frequency (**$\text{Null}$**), uncorrected fixation index (****), fixation index corrected for null alleles (**$F_{\text{null}}$**), per locus and as a mean for all loci for the regenerant population (RP) and populations along highways of MS and SP.**

| Locus |  | Missing data (%) | $k$ |  |  |  | $\text{Null}$ | $F_{\text{null}}$ |
| --- | --- | --- | --- | --- | --- | --- | --- | --- |
| RP |  |  |  |  |  |  |  |  |
| Ga02 | 364 | 5.7 | 15 | 0.777 | 0.867 | 0.104* | 0.008 | 0.093 |
| Ga03 | 379 | 1.8 | 14 | 0.778 | 0.801 | 0.028 | 0.002 | 0.026 |
| Ga04 | 386 | 0 | 15 | 0.775 | 0.843 | 0.081* | 0.002 | 0.078 |
| Ga05 | 386 | 0 | 9 | 0.689 | 0.768 | 0.103* | 0.005 | 0.089 |
| Ga06 | 374 | 3.1 | 14 | 0.738 | 0.819 | 0.099* | 0.010 | 0.093 |
| Ga07 | 381 | 1.3 | 9 | 0.740 | 0.748 | 0.010 | 0.000 | 0.009 |
| Ga08 | 385 | 0.3 | 8 | 0.701 | 0.779 | 0.099* | 0.005 | 0.092 |
| Ga09 | 386 | 0 | 7 | 0.648 | 0.722 | 0.104* | 0.000 | 0.104 |
| Mean | 380.1 | 1.5 | 11.4 | 0.731 | 0.793 | 0.079* | 0.004 | 0.073 |
| MS |  |  |  |  |  |  |  |  |
| Ga02 | 49 | 0 | 16 | 0.900 | 0.913 | 0.014 | 0.019 | -0.004 |
| Ga03 | 49 | 0 | 15 | 0.740 | 0.786 | 0.059 | 0.045 | 0.056 |
| Ga04 | 49 | 0 | 15 | 0.860 | 0.899 | 0.043 | 0.021 | 0.027 |
| Ga05 | 47 | 4.1 | 10 | 0.787 | 0.780 | -0.009 | 0 | -0.009 |
| Ga06 | 46 | 6.2 | 14 | 0.891 | 0.883 | -0.009 | 0 | -0.009 |
| Ga07 | 47 | 4.1 | 10 | 0.809 | 0.800 | -0.010 | 0 | -0.01 |
| Ga08 | 48 | 2.0 | 10 | 0.875 | 0.851 | -0.028 | 0 | -0.028 |
| Ga09 | 49 | 0 | 11 | 0.880 | 0.894 | 0.015 | 0.008 | -0.004 |
| Mean | 48 | 2.0 | 12.6 | 0.843 | 0.851 | 0.009 | 0.012 | 0.002 |
| SP |  |  |  |  |  |  |  |  |
| Ga02 | 79 | 0 | 15 | 0.797 | 0.818 | 0.025 | 0.053 | -0.044 |
| Ga03 | 79 | 0 | 11 | 0.709 | 0.775 | 0.085 | 0.048 | 0.043 |
| Ga04 | 78 | 1.3 | 13 | 0.872 | 0.88 | 0.009 | 0.017 | -0.012 |
| Ga05 | 79 | 0 | 9 | 0.835 | 0.837 | 0.002 | 0.011 | -0.014 |
| Ga06 | 78 | 1.3 | 10 | 0.833 | 0.822 | -0.014 | 0 | -0.014 |
| Ga07 | 79 | 0 | 9 | 0.835 | 0.785 | -0.064 | 0 | -0.064 |
| Ga08 | 79 | 0 | 8 | 0.886 | 0.866 | -0.023 | 0 | -0.023 |
| Ga09 | 79 | 0 | 11 | 0.835 | 0.85 | 0.017 | 0.008 | 0.006 |
| Mean | 78.8 | 0.32 | 10.8 | 0.825 | 0.829 | 0.005 | 0.017 | -0.015 |

$n$ is the sample size; $k$ is the total number of alleles; is the observed heterozygosity; is the expected heterozygosity; *P< 0.05.
