# Supplementary material for: Landscape barriers to pollen and seed flow in the dioecious tropical tree Astronium fraxinifolium in Brazilian savannah
Source: PLoS One. 2021 Aug 2;16(8):e0255275. doi: 10.1371/journal.pone.0255275 (PMC8336915; doi:10.1371/journal.pone.0255275)
Supplement: S3 Table — (DOCX) [file pone.0255275.s004.docx]

Landscape barriers to pollen and seed flow in the dioecious tropical tree *Astronium fraxinifolium* in Brazilian savannah

Ricardo O. Manoel^1^, Bruno C. Rossini^1*^, Maiara R. Cornacini^2^, Mário L. T. Moraes^3^, José Cambuim^3^, Marcelo A. M. Alcântara^2^, Alexandre M. Silva^3^, Alexandre M. Sebbenn^4^, Celso L. Marino^1,2^

^1^Instituto de Biotecnologia/ UNESP, Botucatu, São Paulo, Brazil

^2^Instituto de Biociências/ UNESP, Botucatu, São Paulo, Brazil

^3^Faculdade de Engenharia de Ilha Solteira/ UNESP, Ilha Solteira, São Paulo, Brazil

^4^ **Departamento de Melhoramento e Conservação Genética,** Instituto Florestal de São Paulo, Piracicaba, São Paulo, Brazil

*** Correspondence:**Corresponding Author
[bruno.rossini@unesp.br](about:blank)

**Journal: PLOSONE**

**S3 Table. Results intrapopulacional genetic structure (SGS) of** *Astronium fraxinifolium* **in the regenerant population (RP).**

|  | Distance classes | | | | | | | |
| --- | --- | --- | --- | --- | --- | --- | --- | --- |
| Maximum distance (m) | 10 | 25 | 50 | 75 | 100 | 250 | 400 | 928 |
| Mean distance (m) | 5.3 | 17.6 | 37.4 | 62.8 | 86.9 | 178.9 | 321.1 | 477.3 |
| Number of pairs | 977 | 1926 | 2916 | 3627 | 4098 | 26545 | 24533 | 9683 |
| % participation | 84.7 | 92.5 | 95.3 | 98.4 | 98.7 | 99.7 | 100 | 100 |
| CV participation (%) | 0.93 | 0.87 | 0.58 | 0.61 | 0.62 | 0.35 | 0.29 | 1.05 |
| Mean coancestry:  | 0.078 | 0.043 | 0.026 | 0.009 | 0.007 | -0.0003 | -0.008 | -0.010 |
| : 1.96SE | 0.021 | 0.009 | 0.006 | 0.005 | 0.004 | 0.001 | 0.003 | 0.003 |

 is the mean coancestry coefficient in each distance classes; 1.96SE is the 95% standard error of mean coancestry coefficient; CV is the coefficient of variation.
