# Supplementary material for: Landscape barriers to pollen and seed flow in the dioecious tropical tree Astronium fraxinifolium in Brazilian savannah
Source: PLoS One. 2021 Aug 2;16(8):e0255275. doi: 10.1371/journal.pone.0255275 (PMC8336915; doi:10.1371/journal.pone.0255275)
Supplement: S4 Table — (DOCX) [file pone.0255275.s005.docx]

Landscape barriers to pollen and seed flow in the dioecious tropical tree *Astronium fraxinifolium* in Brazilian savannah

Ricardo O. Manoel^1^, Bruno C. Rossini^1*^, Maiara R. Cornacini^2^, Mário L. T. Moraes^3^, José Cambuim^3^, Marcelo A. M. Alcântara^2^, Alexandre M. Silva^3^, Alexandre M. Sebbenn^4^, Celso L. Marino^1,2^

^1^Instituto de Biotecnologia/ UNESP, Botucatu, São Paulo, Brazil

^2^Instituto de Biociências/ UNESP, Botucatu, São Paulo, Brazil

^3^Faculdade de Engenharia de Ilha Solteira/ UNESP, Ilha Solteira, São Paulo, Brazil

^4^ **Departamento de Melhoramento e Conservação Genética,** Instituto Florestal de São Paulo, Piracicaba, São Paulo, Brazil

*** Correspondence:**Corresponding Author
[bruno.rossini@unesp.br](about:blank)

**Journal: PLOSONE**

**S4 Table. Results of parentage analysis for simulations and observed at strict (95%) and relaxed (80%) critical delta (**$\Delta$**) for individuals of the regenerant population (father or pollen parent; mother and seed parent) for** *Astronium fraxinifolium***.**

|  | Mother alone (assignments) | | | Father alone (assignments) | | | Parent pair: sexes known  (assignments) | | |
| --- | --- | --- | --- | --- | --- | --- | --- | --- | --- |
|  | $\Delta$ | Simulation:  $n$ (%) | Observed:  $n$ (%) | $\Delta$ | Simulation:  $n$ (%) | Observed:  $n$ (%) | $\Delta$ | Simulation:  $n$ (%) | Observed:  $n$ (%) |
| Strict (95%) | 4.56 | 3801 (38) | 44 (11.9) | 4.54 | 3845 (38) | 16 (4.3) | 6.17 | 3494 (35) | 16 (4.3) |
| Relaxed (80%) | 1.49 | 6931 (69) | 159 (43) | 1.49 | 6926 (69) | 87 (23.5) | 3.04 | 5428 (54) | 62 (16.8) |
| Unassigned |  | 3069 (31) | 221 (57) |  | 4074 (31) | 283 (76.5) |  | 4572 (46) | 308 (83.2) |
| Total |  | 10000 | 370 |  | 10000 | 370 |  | 10000 | 370 |

$n$ is the number of individuals.
